# The front line of defence: a meta-analysis of apoplastic proteases in plant immunity

**DOI:** 10.1093/jxb/eraa602

**Published:** 2021-01-19

**Authors:** Alice Godson, Renier A L van der Hoorn

**Affiliations:** 1 The Plant Chemetics Laboratory, Department of Plant Sciences, University of Oxford, Oxford, UK; 2 University of Ljubljana, Slovenia

**Keywords:** Apoplast, defence, hypersensitive response, immunity, pathogen, plant, protease, recognition, signalling

## Abstract

Secreted proteases act at the front line of defence and play pivotal roles in disease resistance. However, the criteria for apoplastic immune proteases are not always defined and followed. Here, we critically reviewed 46 apoplastic proteases that function in plant defence. We found that most apoplastic immune proteases are induced upon infection, and 17 proteases are genetically required for the immune response. Proteolytic activity has been confirmed for most of the proteases but is rarely shown to be required for biological function, and the apoplastic location of proteases can be subjective and dynamic. Pathogen-derived inhibitors have only been described for cysteine and serine proteases, and the selection pressure acting on immune proteases is rarely investigated. We discuss six different mechanisms by which these proteases mediate plant immunity and summarize the challenges for future research.

## Teaser

This meta-analysis discusses 46 known apoplastic immune proteases for their apoplastic location, protease activity, and their contribution to immunity, and identifies six different mechanisms for proteases acting in immunity.

## Introduction

Proteases are present throughout the tree of life, determining the fate of proteins by irreversibly cleaving peptide bonds. This cleavage serves not only to degrade proteins, thereby rendering them non-functional and facilitating protein turnover, but also to activate proteins through the removal of inhibitory or regulatory domains and changing their subcellular location (Van der Hoorn, 2008).

Plant proteases perform critical functions during the interaction between plants and pathogens. Upon pathogen entry, pathogen-associated molecular patterns (PAMPs) such as chitin and flagellin are recognized by pattern recognition receptors (PRRs), resulting in PAMP-triggered immunity (PTI) (Jones and Dangl, 2006). Adapted pathogens use effector proteins to perturb PTI. Resistant plants can recognize some of these effectors through nucleotide-binding leucine-rich repeat (NB-LRR) proteins, resulting in effector-triggered immunity (ETI) (Zipfel, 2014). ETI often culminates in a form of programmed cell death (PCD) known as the hypersensitive response (HR) at the site of infection, limiting the spread of the pathogen (Morel and Dangl, 1997). Both PTI and ETI trigger similar downstream responses, including the release of reactive oxygen species (ROS), activation of mitogen-activated protein kinases (MAPKs), and the production of pathogenesis-related (PR) proteins such as chitinases, glucanases, and proteases (Dodds and Rathjen, 2010). Local defence responses often trigger systemic acquired resistance (SAR), underpinned in part by the signalling hormone salicylic acid (SA).

**Fig. 1.**
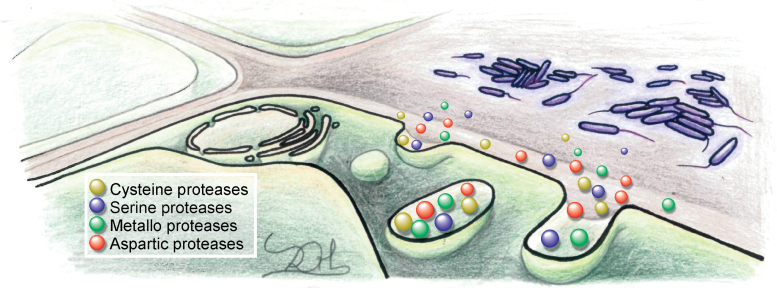
Apoplastic immune proteases at the extracellular battlefield. Proteases of the four major catalytic classes are secreted from the cell into the apoplast, an important site for pathogen colonization. Here, they coordinate the plant immune response through six main strategies: direct antimicrobial activity; immune hydrolase activation; damage-associated molecular pattern (DAMP) release; effector perception; initiation of the hypersensitive response (HR); and the regulation of both systemic acquired resistance and priming.

Whilst much of the plant immune response comprises intracellular events such as transcriptional reprogramming and MAPK signalling, most plant–pathogen interactions occur in the extracellular space known as the apoplast. This is the first and often final destination for pathogens that have entered the plant through wounds, stomata, and hydathodes, and is an important site for pathogen proliferation (Jones and Dangl, 2006). The apoplast can be thought of as an ancient ‘battlefield’ that must be fiercely defended by the host (Jones and Dangl, 2006; Doehlemann and Hemetsberger, 2013; Jashni *et al.*, 2015). Plants are armed with critical weaponry in the form of apoplastic proteases, which they secrete from their cells both constitutively and inducibly (Fig. 1). These proteases are highly stable and active at acidic pH, crucial for their function in the proteolytically challenging environment of the apoplast (Simões and Faro, 2004; Richau *et al.*, 2012). The MEROPS database (Rawlings *et al.*, 2008) provides a classification of proteases into four main groups depending on their catalytic mechanism: aspartic proteases, cysteine proteases, metalloproteases, and serine proteases (Van der Hoorn, 2008).

There are several reviews that discuss the role of proteases in plant immunity (Figueiredo *et al.*, 2014; Misas-Villamil *et al.*, 2016; Balakireva and Zamyatnin, 2018; Thomas and Van der Hoorn, 2018), but a recent comprehensive overview on the role of apoplastic proteases is missing. In this review we will outline the catalytic classes of plant proteases, and then undertake a meta-analysis of the current literature on apoplastic immune proteases. We will finally discuss the various functions these proteases perform to illustrate the critical role apoplastic immune proteases play at multiple stages of the defence response.

## Overview of the catalytic classes of plant proteases

Plants produce 500–1000 proteases, which belong to four main catalytic classes and a few additional catalytic classes (Van der Hoorn, 2008).

Aspartic (Asp) proteases are characterized by the presence of two Asp residues which support a molecule of water that acts as a nucleophile during proteolysis (Van der Hoorn, 2008). Asp proteases are grouped into 14 different families in the MEROPS database (Rawlings *et al.*, 2008), of which five contain plant proteases. The majority of plant Asp proteases belong to the A1 family of pepsin-like proteases (pepsins) (Simões and Faro, 2004). These pepsins may be typical, atypical, or nucellin-like, depending on their active site motif and other sequence features such as the presence of a plant-specific insert called a swaposin (Simões and Faro, 2004). The majority of plant pepsins are intracellular, usually with a vacuolar location, but there are several plant pepsins accumulating in the apoplast (Simões and Faro, 2004).

Cysteine (Cys) proteases utilize a catalytic Cys residue in their catalytic triad as a nucleophile during proteolysis (Van der Hoorn, 2008). Plant Cys proteases are represented in five MEROPS clans (Rawlings *et al.*, 2008). Papain-like cysteine proteases (papains) are the only family containing extracellular Cys proteases and have long been implicated in plant immunity (Misas-Villamil *et al.*, 2016). Papains belong to MEROPS clan CA, family C1 and are grouped into nine subfamilies (Richau *et al.*, 2012). The general papain structure consists of a papain-like fold of an α-helix and β-sheet domain, and two papain subfamilies also carry a C-terminal granulin domain. Being very stable enzymes with multiple disulfide bridges, papains are often found in proteolytically harsh environments, such as the apoplast and vacuole (Richau *et al.*, 2012). Their apoplastic location puts papains in direct contact with pathogens and their associated proteins, making them prime candidates for involvement in the plant immune response.

Metalloproteases utilize a catalytic metal cofactor to activate water that acts as a nucleophile during proteolysis (Van der Hoorn, 2008). There are >50 families of metalloproteases in the MEROPS database (Rawlings *et al.*, 2008). Plants have representatives that belong to 19 families, which are grouped into 11 evolutionarily unrelated clans (Van der Hoorn, 2008). The matrix metalloproteases (MMPs) of family M10 are the only plant metalloprotease family with apoplastic proteases. MMPs are zinc-dependent endopeptidases that share a conserved catalytic domain containing a zinc-binding sequence and a conserved Met residue that forms a ‘Met-turn’ (Marino and Funk, 2012). All plant MMPs contain a signal peptide and are either secreted into the plant apoplast or anchored to the plasma membrane (Flinn, 2008; Marino and Funk, 2012).

Serine (Ser) proteases are the largest class of proteases in plants and are unified by the use of Ser in the active site as a nucleophile (Van der Hoorn, 2008). Plants contain 14 Ser protease families which are grouped into nine evolutionarily unrelated clans. Subtilisin-like proteases (subtilases) of subfamily S8A are often secreted and are the most extensively studied (Schaller *et al.*, 2018). Serine carboxypeptidase-like proteins (SCPLs) are found in MEROPS subfamily S10 and comprise another abundant serine hydrolase subfamily (Van der Hoorn *et al.*, 2011). Whilst many SCPLs have carboxypeptidase activity (Li *et al.*, 2001; Casamitjana-Martínez *et al*., 2003), some catalyse transacylation reactions rather than cleaving C-terminal peptide bonds (Mugford and Milkowski, 2012). All Arabidopsis SCPLs contain signal peptides targeting them for secretion (Fraser *et al.*, 2005) and many SCPLs are detected in the apoplast (Sueldo *et al.*, 2014; Grosse-Holz *et al.*, 2018).

The MEROPS database divides proteases into a further three smaller classes, in addition to the four major classes discussed above. These are Asn peptide lyases, Glu proteases, and Thr proteases. Asn peptide lyases and Glu proteases have not been studied extensively and plant members are yet to be identified (Rawlings *et al.*, 2011). Thr proteases such as Arabidopsis PBA1 form subunits of the 26S proteasome that mediates protein degradation (Tanaka, 2009). PBA1 has caspase-3-like activity and is involved in the induction of PCD upon infection with avirulent bacteria (Hatsugai *et al.*, 2009), but is not an apoplastic immune protease. These three protease classes are not further discussed in this review because they do not contain apoplastic immune proteases.

## Meta-analysis of apoplastic immune proteases

Scientific advances have identified and characterized an increasing number of apoplastic immune proteases. We undertook a meta-analysis of proteases that are both apoplastic and involved in immunity. We identified 46 putative apoplastic immune proteases, summarized in Table 1. For the purpose of the analysis, we counted orthologues of a gene in different species separately. Closely related paralogues from the same species that show indistinguishable immune phenotypes or act redundantly, *CathB1*, *-B2*, and *-B3* in Arabidopsis for instance (McLellan *et al.*, 2009), are counted only once.

**Table 1.** Criteria for apoplastic immune proteases

Name	Criteria:		A	B1	B2	B3	B4	B5	C1	C2	Reference
	MEROPS Family	Organism	Apoplastic?	Genetically required?	Induced?	Inhibited?	Diversifying selection?	Secretion prevented?	Protease?	Activity required?	
AED1	A01	*At*	Y	–	Y	–	–	–	–	–	Breitenbach *et al.* (2014)
*Gm*AP1	A01	*Gm*	Y	Y	–	–	–	Y	Y	Y	Guo *et al.* (2019)
*St*AP1/3	A01	*St*	Y	–	Y	–	–	–	Y	–	Guevara *et al*. (1999, 2001, 2002, 2004); Mendieta *et al.* (2006); Muñoz *et al.* (2010)
*Os*AP77	A01	*Os*	–	Y	Y	–	–	–	–	–	Alam *et al.* (2014)
*Le*AspP	A01	*Sl*	–	–	Y	–	–	–	–	–	Schaller and Ryan (1996)
CDR1	A01	*At*	Y	Y	Y	–	–	–	Y	Y	Xia *et al.* (2004); Simões *et al.* (2007)
		*Os*	Y	–	Y	–	–	–	Y	Y	Prasad *et al.* (2009)
SAP1/2	A01	*At*	Y	Y	Y	–	–	–	Y	Y	Wang *et al.* (2019)
C14	C1A	*Sl*	Y	–	Y	Y	**N**	Y	Y	–	Harrak *et al.* (2001); Kaschani *et al.* (2010); Bozkurt *et al.* (2011); Kovács *et al.* (2016); Shindo *et al.* (2016)
		*St*	-	–	Y	–	Y	–	–	–	Avrova *et al.* (1999); Kaschani *et al.* (2010); Kaschani and Van der Hoorn (2011)
CathB	C1A	*At*	Y*	Y	Y	–	–	–	Y	–	McLellan *et al.* (2009); Ge *et al.* (2016); Porodko *et al.* (2018)
		*Nb*	Y	Y	Y	–	–	–	Y	–	Gilroy *et al.* (2007)
		*St*	–	–	Y	–	–	–	–	–	Avrova *et al.* (2004)
CP1/2	C1A	*Zm*	Y	–	Y	Y	–	–	Y	–	Mueller *et al.* (2013); Ziemann *et al.* (2018); Misas Villamil *et al.* (2019)
CP14	C1A	*Nb*	Y*	Y	–	–	–	–	Y	–	Kaschani *et al.* (2010); Bozkurt *et al.* (2011); Paireder *et al.* (2016)
Mir1	C1A	*Zm*	Y	–	Y	Y	–	–	Y	Y	Pechan *et al*. (2000, 2002); Mohan *et al.* (2006); Lopez *et al.* (2007); Fescemyer *et al.* (2013); Louis *et al.* (2015); Varsani *et al.* (2016)
MRP1/2	C1A	*Mj*	Y	–	–	Y	–	–	Y	–	Dong *et al.* (2014)
Papain	C1A	*Cp*	Y	–	Y	Y	–	–	Y	–	El Moussaoui *et al.* (2001); Konno *et al.* (2004); Azarkan *et al.* (2006); Gumtow *et al.* (2018)
Pip1	C1A	*Sl*	Y	Y	Y	Y	–	–	Y	–	Tian *et al.* (2007); Shabab *et al.* (2008); Ilyas *et al.* (2015); Shindo *et al.* (2016); Planas-Marquès *et al.* (2018)
*Cs*RD21a	C1A	*Cs*	Y	–	Y	Y	–	–	Y	–	Clark *et al.* (2018)
Rcr3	C1A	*Sl*	Y	Y	Y	Y	Y	–	Y	–	Dixon *et al.* (2000); Krüger *et al.* (2002); Rooney *et al.* (2005); Shabab *et al.* (2008); Song *et al.* (2009); Lozano-Torres *et al.* (2012); Dong *et al.* (2014); Ilyas *et al.* (2015); Shindo *et al.* (2016); Paulus *et al.* (2020)
*Cs*SAG12-1	C1A	*Cs*	–	–	Y	–	–	–	Y	–	Clark *et al.* (2018)
XCP2	C1A	*Os*	–	Y	Y	–	–	–	–	–	Niño *et al.* (2020)
		*Zm*	Y	–	Y	Y	–	–	Y	–	van der Linde *et al.* (2012); Mueller *et al.* (2013)
*At*-MMPs	M10	*At*	Y	Y	Y	–	–	–	Y	–	Maidment *et al.* (1999); Marino *et al.* (2014); Zhao *et al.* (2017)
*Gm*MMP2	M10	*Gm*	–	–	Y	–	–	–	Y	–	Liu *et al.* (2001)
*Nt*MMP1	M10	*Nt*	Y	–	Y	–	–	–	Y	–	Schiermeyer *et al.* (2009); Mandal *et al.* (2010)
*Sl*-MMPs	M10	*Sl*	Y	Y	Y	–	–	–	Y	–	Li *et al.* (2015); Zimmermann *et al.* (2016)
*Hv*PR-17a/-17b	M10?	*Hv*	Y	–	Y	–	–	–	–	–	Christensen *et al.* (2002)
*Nb*PRp27	M10?	*Nb*	–	Y	Y	–	–	–	–	–	Xie and Goodwin (2009)
*Nt*PRp27	M10?	*Nt*	Y	–	Y	–	–	–	–	–	Okushima *et al.* (2000)
WCI-5	M10?	*Ta*	–	–	Y	–	–	–	–	–	Görlach *et al.* (1996); Schweizer *et al.* (1999)
*Os*BISCPL1	S10	*Os*	–	–	Y	–	–	–	–	–	Liu *et al.* (2008)
P69B	S8	*Sl*	Y	–	Y	Y	–	–	Y	Y	Granell *et al.* (1987); Tornero *et al.* (1997); Jordá *et al.* (1999); Zhao *et al.* (2003); Tian *et al*. (2004, 2005); Zimmermann *et al.* (2016); Planas-Marquès *et al.* (2018); Wang *et al.* (2020); Paulus *et al.* (2020)
P69C	S8	*Sl*	Y	–	Y	–	–	–	Y	–	Tornero *et al.* (1996); Jordá *et al.* (1999); Planas-Marquès *et al.* (2018)
*Sl*Phyt-1/-2	S8	*Sl*	Y	–	–	–	–	–	Y	–	Beloshistov *et al.* (2018); Reichardt *et al.* (2018)
Phytaspase	S8	*Nt*	Y	Y	Y	–	–	–	Y	–	Chichkova *et al*. (2004, 2010)
		*Os*	–	–	–	–	–	–	Y	Y	Chichkova *et al.* (2010)
SAS-1/-2	S8	*As*	Y	–	**N**	–	–	–	Y	–	Coffeen and Wolpert (2004)
*Gb*SBT1	S8	*Gb*	Y	Y	Y	–	–	–	–	–	Duan *et al.* (2016(
SBT3	S8	*Sl*	Y	Y	Y	–	–	–	Y	–	Meyer *et al*. (2016a, b)
SBT3.3	S8	*At*	Y	Y	Y	–	–	–	Y	Y	Ramírez *et al.* (2013)
SBT3.5	S8	*At*	Y	–	–	–	–	–	Y	–	Bethke *et al.* (2014); Sénéchal *et al.* (2014)
*Nb*SBT5.2	S8	*Nb*	Y	–	–	–	–	–	Y	–	Wang *et al.* (2020) Paulus *et al.* (2020)
*S*tSBTc-3	S8	*St*	Y	–	Y	–	–	–	Y	Y	Fernández *et al*. 2012, 2015)
*Hb*SPA	S8	*Hb*	Y	–	Y	Y	–	–	Y	–	Ekchaweng *et al.* (2017)

A total of 46 proteases are evaluated against the ABC criteria for apoplastic immune proteases: (A) apoplastic location; (B) biological function in immunity; and (C) catalytic activity as a protease. *As*, *Avena sativa; At*, *Arabidopsis thaliana*; *Cp*, *Carica papaya; Cs*, *Citrus sinensis*; *Gb*, *Gossypium babardense*; *Gm*, *Glycine max*; *Hb*, *Hevea brasiliensis; Hv*, *Hordeum vulgare*; *Mj*, *Mirabilis jalapa*; *Nb*, *Nicotiana benthamiana*; *Nt*, *Nicotiana tabacum*; *Os*, *Oryza sativa*; *Sl*, *Solanum lycopersicum*; *St*, *Solanum tuberosum*; *Ta*, *Triticum aestivum; Zm*, *Zea mays*; Y (Yes), evidence that protein meets criteria; **N** (No), evidence that protein does not meet criteria; – protein not tested against criteria; *not shown *in planta*.

To qualify as an apoplastic immune protease, experimental evidence is required for each of these three defining words: apoplastic, immune, and protease. This can be summarized as the ABC criteria: (A) apoplastic location; (B) biological function in immunity; and (C) catalytic activity as a protease. We critically evaluated each of the 46 putative apoplastic immune proteases for fulfilling these criteria, summarized in Table 1 and discussed below.

### Criterion A (apoplast): apoplastic location can be subjective and dynamic

In total, 35 of the 46 identified proteins (76%) have been shown to be secreted, although whether a protein is considered apoplastic is dependent on definition. We consider a protease to be apoplastic if its catalytic domain is located outside of the plasma membrane. This encompasses the extracellular space and the xylem, but also the cell wall and latex. The picture is complicated when considering that proteins are often found in multiple locations within and outside the cell. The papain C14, for example, is found in the vacuoles, vesicles, endoplasmic reticulum, and apoplast of tomato (Bozkurt *et al.*, 2011).

The literature is abounding with examples of proteases whose apoplastic location is predicted on the basis of the extracellular location of orthologous proteases, or the use of prediction software such as ApoplastP, which utilizes machine learning to predict the location based on amino acid enrichment and depletion patterns (Sperschneider *et al.*, 2018), and SignalP, which predicts signal peptides and their cleavage sites (Almagro Armenteros *et al.*, 2019). These methods of prediction are not infallible, and experimental evidence is usually obtained to confirm the location of the protease. This might include detection of the protease from apoplastic fluid or confocal microscopy with fluorescent fusion proteins.

Protein location is also dynamic, and both import to and export from the apoplast are inducible upon both abiotic and biotic stress. Proteases are released from the vacuole into the apoplast upon PCD induced by avirulent bacteria (Hatsugai *et al.*, 2009). Conversely, phytaspase is constitutively secreted into the apoplast of tobacco and rice before being reimported into the cell upon PCD (Chichkova *et al.*, 2010). Similarly, cotton subtilase *Gb*SBT1 relocates from the apoplast to the cytoplasm during defence against the fungus *Verticillium dahliae* (Duan *et al.*, 2016). Extracellular *Gb*SBT1 interacts with the secreted *V. dahliae* effector prohibitin, which may trigger the movement of *Gb*SBT1 into the cell (Duan *et al.*, 2016).

### Criterion B (biological role)

#### B1: 17 proteases are genetically required for immunity

Genetic requirement for pathogen resistance has been demonstrated for 17 of the 46 identified proteases (37%). For example, antisense *Pip1* tomato plants are highly susceptible to *Cladosporium fulvum*, *Pseudomonas syringae*, and *Phytophthora infestans* (Ilyas *et al.*, 2015). Likewise, hairpin silencing of tomato *SBT3* increases the growth of *Manduca sexta* larvae (Meyer *et al.*, 2016a), and depletion of *NbPRp27* by virus-induced gene silencing (VIGS) increases susceptibility to *P. syringae* pv. *tabaci* (Xie and Goodwin, 2009).

In contrast to the 17 confirmed proteases contributing positively to immunity, the APOPLASTIC, EDS1-DEPENDENT 1 (AED1) Asp protease in Arabidopsis might be suppressing immune responses (Breitenbach *et al.*, 2014). *AED1* silencing results in elevated transcripts of the SAR marker gene *PR1*, as well as severe stunting, indicative of a constitutive defence phenotype. However, disease assays showing significantly increased resistance of silenced plants compared with wild-type plants have not yet been reported.

Redundancy amongst closely related immune proteases can make it difficult to untangle immune functions. For instance, studies on five MMP genes in Arabidopsis showed that triple mutants had a stronger defect in immunity when compared with single mutants to both *Botrytis cinerea* and *Golovinomyces orontii* (Zhao *et al.*, 2017). Similarly, Arabidopsis *CathB1*, -*B2*, and -*B3* show high sequence identity and are redundantly involved in basal resistance and the development of PCD (McLellan *et al.*, 2009).

Genetic requirement should be demonstrated through knockdown or knockout of the gene in question, and cannot be inferred from lines that naturally accumulate different levels of the protease. For instance, the protease Mir1 accumulates in resistant but not in susceptible maize lines, and there is a negative correlation between Mir1 protein concentration and larval weight (Pechan *et al.*, 2000). However, this cannot be used as evidence for the genetic requirement for Mir1 since there are likely to be other differences between these lines. Overexpression of a protease is also used to infer an immune function, but these experiments must also be interpreted with caution since they do not demonstrate the endogenous role of the protease.

#### B2: protease induction at the transcript/protein level is common

In total, 38 of the 46 proteases (83%) are induced at the transcript and/or protein level. This suggests that nearly all immune proteases are PR proteins, or alternatively that proteases that are not induced, but do function in immunity, are being overlooked. For instance, mRNA corresponding to the tomato Asp protease *Le*AspP is induced in leaves upon wounding (Schaller and Ryan, 1996), and there is a rapid accumulation of soybean *GmMMP2* transcripts upon infection with *Phytophthora sojae* and *P. syringae* pv. *glycinea* (Liu *et al.*, 2001). Likewise, experiments with coffee cultivars showed an increase in apoplastic Ser protease activity upon *Hemileia vastratrix* infection, particularly in resistant cultivars (Guerra-Guimarães *et al.*, 2015), and activity-based protein profiling (ABPP; explained in section C1) uncovered an increased activity of Cys and Ser proteases Pip1, P69B, and P69C in tomato upon *Ralstonia solanacearum* infection (Planas-Marquès *et al.*, 2018). However, not all immune proteases are induced. The oat subtilases SAS-1 and SAS-2 are constitutively transcribed and present in the cell, but apoplastic activity is induced by relocation into the apoplast at the onset of PCD induced by the fungal toxin victorin (Coffeen and Wolpert, 2004).

#### B3: identification of pathogen-derived inhibitors has been restricted to Cys and Ser proteases

Inhibition by pathogen-derived inhibitors has been described for 11 of the 46 immune proteases (24%). Notably, nine of these 11 proteases are Cys proteases. In fact, the Cys proteases Rcr3, Pip1, and C14 are each targeted by multiple inhibitors (Tian *et al.*, 2007; Shabab *et al.*, 2008; Kaschani *et al.*, 2010; Lozano-Torres *et al.*, 2012; Shindo *et al.*, 2016), and the same inhibitor often targets multiple Cys proteases (Clark *et al.*, 2018). The concept of adaptation of a pathogen and its inhibitor repertoire to its host is neatly demonstrated by the case of *Phytophthora mirabilis*, an oomycete closely related to *P. infestans*, which infects the four-o’clock flower (*Mirabilis jalapa*). Whilst EPIC1 of *P. infestans* inhibits *Solanum* Rcr3, *Pm*EPIC1 of *P. mirabilis* has specialized to inhibit the Rcr3-related proteases MRP1 and MRP2 of *M. jalapa*. The specialization of each effector to its corresponding protease was underpinned by a single amino acid polymorphism in the host protease along with a reciprocal single amino acid change in the pathogen effector (Dong *et al.*, 2014).

Despite the bias in the literature towards the identification of Cys protease inhibitors, there are also some known examples of Ser protease inhibitors. Tomato P69B is inhibited by two Kazal-like Ser protease inhibitors of *P. infestans*, EPI1 and EPI10 (Tian *et al*., 2004, 2005). These inhibitors are distinct, differing in the number and sequence of the Kazal-like domains they possess. Inhibition of P69B by two divergent inhibitors suggests that this is an important infection strategy for the pathogen. The *Phytophthora palmivora* homologue of EPI10 is secreted into the apoplast during infection of the rubber tree (*Hevea brasiliensis*) (Chinnapun *et al.*, 2009) and inhibits the apoplastic subtilase *Hb*SPA, which may otherwise mediate resistance to the pathogen (Ekchaweng *et al.*, 2017). Whilst inhibitors of apoplastic Asp and metalloproteases have not yet been reported, there is no inherent reason why they could not be targeted by pathogen-derived inhibitors.

#### B4: selection pressure acting on immune proteases is rarely investigated

Only two of the 46 immune proteases (4%) have been shown to be under diversifying selection: Rcr3 and *St*C14 (Shabab *et al.*, 2008; Kaschani *et al.*, 2010; Kaschani and Van der Hoorn, 2011). Their variant residues locate around the substrate-binding site as a footprint of an arms race with pathogen-derived inhibitors. The positions of the variant residues in Pip1 and P69B are also consistent with the presence of diversifying selection caused by pathogen-derived inhibitors (Shabab *et al.*, 2008; Hörger and Van der Hoorn, 2013). This low number is not because of negative results for the remaining proteases, but rather because this aspect has not been investigated for most immune proteases. Only *Sl*C14 is confirmed to be under stabilizing, rather than diversifying, selection (Shabab *et al.*, 2008). The difference in selection pressure between tomato and potato C14 probably reflects the specialization of *P. infestans* to wild potato, its natural host (Kaschani *et al.*, 2010).

#### B5: two proteases are prevented from being secreted into the apoplast

The prevention of protease secretion into the apoplast has been identified for two of the 46 proteases (4%). Secretion of the tomato Cys protease C14 is prevented by the *P. infestans* RxLR effector AvrBlb2, which accumulates around haustoria. An *AvrBlb2* mutant impaired in haustorial localization allowed apoplastic C14 accumulation and reduced the growth of *P. infestans* (Bozkurt *et al.*, 2011). Likewise *P. sojae*, the cause of soybean stem and root rot, secretes the plasma membrane-localized RxLR-type effector *Ps*Avr240 that prevents the secretion of the soybean Asp protease *Gm*AP1 into the apoplast (Guo *et al.*, 2019). *Gm*AP1 positively contributes to resistance against *Phytophthora* species, and so, by preventing its secretion, *Gm*AP1-mediated defence is compromised. In both instances, however, the underlying molecular mechanism is unknown.

### Criterion C (catalytic activity)

#### C1: protease activity is shown for most candidates

Protease activity has been confirmed for 34 of the 46 identified proteases (74%). In particular, catalytic activity has been confirmed for the majority of the identified Cys proteases (81%). This probably reflects the relatively thorough characterization of papains and the tools available to monitor their activity.

Catalytic activity is demonstrated by the ability of a protease to degrade a biological or commercial substrate. For instance, gelatin and casein are general protease substrates that were used to confirm the protease activity of tomato Rcr3 and potato *St*SBTc-3, respectively (Krüger *et al.*, 2002; Fernández *et al.*, 2015). Similarly, myelin basic protein and Z-Leu-Arg-MCA have confirmed the catalytic activity of soybean metalloprotease *Gm*MMP2 (Liu *et al.*, 2001) and *Nicotiana benthamiana* Cys protease CP14 (Paireder *et al.*, 2016), respectively. Protease activity can also be inferred by ABPP. This technique uses chemical probes that mimic substrates but covalently bind to the active site of proteases as a readout for their activity (Morimoto and van der Hoorn, 2016). For example, ABPP labelling was used to infer the catalytic activity of *Citrus sinensis* RD21a (Clark *et al.*, 2018), tomato P69C (Planas-Marquès *et al.*, 2018),and *Nb*SBT5.2 (Paulus *et al.*, 2020). Finally, catalytic activity is implicit if criterion C2 is satisfied, even if the activity has not been shown directly—for instance, an active site mutant of soybean Asp protease *Gm*AP1 no longer confers resistance to *Phytophthora capsici*, indicating that the wild-type protease must display catalytic activity (Guo *et al.*, 2019).

#### C2: the requirement of catalytic activity for biological function is rarely shown

The active site of proteases has been shown to be required for the biological function of only nine of the 46 immune proteins (20%). The requirement for catalytic activity can primarily be shown by a catalytically inactive mutant no longer being able to perform the described immune function. For instance, catalytic mutants of Asp protease SAP1 are no longer able to suppress *P. syringae* growth (Wang *et al.*, 2019). Similarly, tobacco silenced for PCD-promoting phytaspase show an abolished *Tobacco mosaic virus* (TMV)-induced HR that can be restored by complementation with wild-type rice phytaspase, but not its catalytic mutant (Chichkova *et al.*, 2010).

Alternatively, the requirement for catalytic activity can be shown through the use of an inhibitor preventing the biological function of the protease. However, this can only be taken as evidence if only the protease in question is being inhibited. Since most commercial inhibitors are not specific to a single protease, the protease must be purified and inhibition studied *in vitro*. For instance, papain itself is released into the latex of papaya upon wounding, where it provides a strong toxicity and growth inhibition against a range of lepidopteran pests (Konno *et al.*, 2004). Papaya leaves painted with the papain inhibitor E-64 support significantly greater larval growth, but this is not proof of the catalytic requirement of papain itself since E-64 may also inhibit other papain-like Cys proteases present in latex (Konno *et al.*, 2004).

Likewise, the antimicrobial activity of extracellular Asp proteases *St*AP1 and *St*AP3 was demonstrated by *in vitro* experiments showing the inhibition of *P. infestans* cyst and *Fusarium solani* conidia germination upon incubation with the purified proteases (Guevara *et al*., 2002, 2004). This antimicrobial activity can be abolished by the addition of the Asp protease inhibitor pepstatin A to the purified proteases (Guevara *et al*., 2002, 2004). Addition of pepstatin A to apoplastic fluid also increases susceptibility to *F. solani*, but this is not enough evidence that these proteases have antimicrobial activity *in vivo*, and that catalytic activity is required for this.

## Six functions of secreted immune proteases

The molecular mechanism by which apoplastic proteases confer immunity appears resolved in eight cases: *St*AP1/3, SAP1/2, Mir1, papain, Rcr3, P69B, *Sl*Phyt-1/-2, and SBT3.5, although genetic requirement for the immune response has not yet been demonstrated for *St*AP1/3, papain, and *Sl*Phyt-1/-2. We divide these mechanisms into four groups: antimicrobial activity, immune hydrolase activation, damage-associated molecular pattern (DAMP) release, and effector perception. A single protease may act on multiple substrates and hence perform multiple functions, as is the case for tomato P69B (Paulus *et al.*, 2020; Wang *et al.*, 2020).

### Apoplastic proteases display direct antimicrobial activity

Apoplastic proteases are in a perfect place to directly damage the pathogen, impeding growth and proliferation (Konno *et al.*, 2004; Mendieta *et al.*, 2006). One of the first papains to be characterized, Mir1 from maize has direct antimicrobial activity against insect pests. This was first indicated by growth inhibition of fall armyworm and tobacco budworm larvae feeding on callus expressing *Mir1* (Pechan *et al.*, 2000). Direct antimicrobial activity was later demonstrated by a sharp increase in the permeability of *Spodoptera frugiperda* peritrophic matrix to Blue Dextran upon the addition of purified Mir1 (Mohan *et al.*, 2006). Mir1 accumulates at the wound site within 1 h of larval feeding, assisted by the protease’s movement through the plant vascular tissues (Lopez *et al.*, 2007). The protease inhibits larval growth by degrading insect intestinal mucin (IIM), which cross-links the chitin fibrils in the peritrophic matrix (Fescemyer *et al.*, 2013). This induces IIM permeabilization, which impairs digestion and nutrient absorption (Pechan *et al.*, 2002; Mohan *et al.*, 2006). Larvae compensate for this damage by altering their midgut transcriptome to up-regulate IIM replacement components (Fescemyer *et al.*, 2013) and produce Cys protease inhibitors in the midgut (Li *et al.*, 2009). Mir1 also provides enhanced resistance to corn leaf aphids above-ground and to western corn rootworm below-ground (Louis *et al.*, 2015; Varsani *et al.*, 2016).

Direct antimicrobial activity has also been shown for secreted aspartic protease 1 and 2 (SAP1 and SAP2) in Arabidopsis. Purified SAP1 and SAP2 are capable of suppressing *P. syringae* growth *in vitro*, demonstrating their antimicrobial activity (Wang *et al.*, 2019). SAP1 and SAP2 contribute redundantly to defence against *P. syringae* through the cleavage of the bacterial protein MucD, which is required for bacterial growth (Wang *et al.*, 2019). Cleavage of MucD by SAP1 and SAP2 suppresses bacterial growth without causing bacterial death. *SAP* homologues are found throughout the Brassicaceae family as well as in tomato and rice, suggesting that this role in immunity could be evolutionarily conserved (Wang *et al.*, 2019).

### Apoplastic proteases activating immune hydrolases

Apoplastic proteases are able to activate other immature hydrolases by removing inhibitory prodomains. For instance, tomato subtilase P69B cleaves after Asp residues to remove the prodomain of Rcr3, thereby activating this immune protease (Paulus *et al.*, 2020). *Nicotiana benthamiana* subtilase SBT5.2 can also process proRcr3 into mature Rcr3, suggesting that activation of immune proteases by subtilases is common in Solanaceous plants (Paulus *et al.*, 2020).

Likewise, Arabidopsis subtilase SBT3.5 activates pectin methylesterases (PMEs) such as PME17 (Sénéchal *et al.*, 2014). PMEs are found in the apoplast and are responsible for the demethylation of homogalacturonan, the major constituent of pectin in the cell wall. Altering the properties of the cell wall can have dramatic consequences for the resistance of plants to pathogens, and there is evidence of both positive and negative roles for PMEs in immunity (Lionetti *et al.*, 2007; Körner *et al.*, 2009). PME17 is required for immunity because its transcripts are up-regulated in response to *P. syringae* and *Alternaria brassicicola* infection, and *pme17* mutants are more susceptible to *P. syringae* (Bethke *et al.*, 2014). This implicates SBT3.5 in immunity through the activation of a cell wall-modifying enzyme.

### Apoplastic proteases mediating DAMP release

Another function of apoplastic proteases is mediating the release of peptides that act as DAMPs (Hou *et al.*, 2019). Tomato phytaspases *Sl*Phyt-1 and *Sl*Phyt-2 can process the defence peptide systemin from its precursor, prosystemin (Beloshistov *et al.*, 2018). Systemin release is required for wound signalling, which is critical in the response to herbivory (Savatin *et al.*, 2014). Prosystemin is found in the cell cytoplasm whilst phytaspases are apoplastic (Chichkova *et al.*, 2010). Therefore, the processing of prosystemin can occur only upon wounding, when cellular integrity is disrupted and the proteins can interact (Beloshistov *et al.*, 2018). The relocalization of phytaspases into the cell during PCD (Chichkova *et al.*, 2010) could also trigger systemin activation, providing a link between PCD and the systemic wound response in tomato (Ryan, 2000).

Maize papains are required for the processing of the propeptide Prozip1 to release Zip1, a small peptide DAMP that activates SA signalling (Ziemann *et al.*, 2018). Zip1 release induces papain activation, thus establishing a positive feedback loop, and promotes SA-mediated defence responses including the up-regulation of defence-related genes such as those encoding chitinases and β-1,3-glucanases, and the mitigation of infection by biotrophic fungi (Ziemann *et al.*, 2018). However, the individual protease responsible for Prozip1 processing has not yet been identified.

### Apoplastic proteases perceive pathogen effectors

Two apoplastic immune proteases act in the perception of pathogen effectors. The tomato papain-like Rcr3 is critical for recognition of the pathogen effector Avr2 produced by the fungus *C. fulvum*. The complex formed when Avr2 inhibits Rcr3 is perceived by the LRR receptor-like protein Cf2, triggering the HR (Rooney *et al.*, 2005). Rcr3 is essential for Avr2 perception and, in the absence of Cf2, Rcr3 does not contribute to immunity to *C. fulvum*, suggesting that it acts as a decoy mimicking the more abundant Rcr3 paralogue Pip1 (Ilyas *et al.*, 2015). However, Rcr3 contributes to *P. infestans* resistance in the absense of Cf2, indicating also a direct role in immunity (Ilyas *et al.*, 2015). Like *C. fulvum*, *P. infestans* secretes effectors to inhibit Rcr3 and other papains. Yet unlike Avr2, EPIC1 and EPIC2B are ‘stealthy’ effectors that inhibit host proteases without being detected by Cf2 (Song *et al.*, 2009).

The second protease involved in effector perception utilizes a very different mechanism. The Cys-rich secreted protein PC2 produced by *P. infestans* is cleaved by apoplastic subtilases including P69B, triggering an immune response including cell death, the accumulation of ROS, and the up-regulation of defence-related genes (Wang *et al.*, 2020). This cleavage is essential for the PC2-triggered immune response and presumably produces a small peptide that is recognized at the cell surface. Interestingly, *P. infestans* is able to inhibit PC2 cleavage and therefore cell death by producing Ser protease inhibitors such as EPI1 (Wang *et al.*, 2020).

### Apoplastic proteases required for the HR

Several apoplastic proteases contribute to HR, although relatively little is understood about the mechanisms by which they regulate and initiate this process. For instance, overexpression and silencing of tobacco phytaspase results in TMV-induced lesions that are larger or smaller, respectively, than those of wild-type plants, providing evidence of a clear involvement of phytaspase in the HR. How this is achieved at the mechanistic level has not yet been elucidated (Chichkova *et al.*, 2010).

The first subtilases associated with PCD, SAS-1 and SAS-2, were identified in 2004 and have caspase-6-like activity. These saspases are thought to be involved in a signalling cascade leading to the cleavage of Rubisco (Coffeen and Wolpert, 2004). Similarly, increased caspase-3-like activity is implicated in the production of necrotic lesions in potato leaves that restrict *P. infestans* growth (Fernández *et al.*, 2012). This activity was later attributed to potato subtilase *St*SBTc-3 which was isolated from *P. infestans*-infected leaves (Fernández *et al.*, 2015). A role in PCD for *St*SBTc-3 is further suggested by the ability of the purified protease to induce cytoplasmic shrinkage and decrease the viability of tomato cell cultures *in vitro* (Fernández *et al.*, 2015). Likewise, the tomato phytaspases *Sl*Phyt-2, -3, -4, -5, and -6 were able to trigger cell death, observed by trypan blue staining, when overexpressed in tomato leaves (Reichardt *et al.*, 2018).

Cathepsin B (CathB) is a papain implicated in PCD in both plants and animals. Silencing of *CathB* in *N. benthamiana* restricts HR triggered by both *Erwinia amylovora* and *P. syringae*, compromising resistance (Gilroy *et al.*, 2007). In Arabidopsis, three *CathB* genes have caspase-3-like activity and contribute redundantly to HR (McLellan *et al.*, 2009; Ge *et al.*, 2016). However, the importance of CathB in HR appears to be context dependent. For instance, *Nb*CathB induces HR triggered by the Avr/R combination Avr3a/R3a, but is not required for HR triggered by Avr4/Cf4 (Gilroy *et al.*, 2007). Likewise, *AtCathB* mutants are more susceptible to virulent *P. syringae*, yet, despite a reduction in AvrB/RPM1-mediated HR, *AtCathB* mutant plants are not compromised in reducing bacterial growth (McLellan *et al.*, 2009). In spite of the evidence that CathB participates in HR regulation, the location of CathB action—apoplastic or intracellular—remains unclear. Studying the role of proteases during the HR is challenged by the fact that protease location cannot be resolved whilst the cell is undergoing PCD.

### Apoplastic proteases mediate SAR and priming

Three apoplastic proteases (AED1, CDR1, and SBT3.3) are involved in signalling leading to local and systemic defence responses, as well as priming the plant for future pathogen invasion. In each case, the substrates of the immune proteases and their position within the signalling cascade are not known.

Constitutive Disease Resistance 1 (CDR1) is an ‘atypical’ Arabidopsis pepsin-like Asp protease identified by T-DNA activation tagging (Xia *et al.*, 2004). *CDR1* overexpression results in increased resistance to virulent *P. syringae* as well as a phenotype mimicking constitutive SAR activation, whilst antisense *CDR1* lines show enhanced bacterial susceptibility (Xia *et al.*, 2004). Upon pathogen invasion, CDR1 accumulates in the apoplast, where it induces both local and systemic defence responses in an SA-dependent manner. Activation of the systemic defence response relies on a mobile elicitor present in extracellular fluids and thought to be generated by CDR1, but its identity is still unknown (Xia *et al.*, 2004). Likewise, rice *OsCDR1* induces the expression of defence-related genes and enhances disease resistance to multiple pathogens when overexpressed in both rice and Arabidopsis (Prasad *et al.*, 2009). Local *OsCDR1* expression in Arabidopsis induces a systemic defence response, suggesting that CDR1 has a conserved function in SA-mediated disease resistance (Prasad *et al.*, 2009). The contrasting roles for CDR1 and AED1 (Criterion B1) raise fascinating questions regarding their evolution and substrate selectivity.

Arabidopsis subtilase SBT3.3 is involved in the regulation of immune priming (Ramírez *et al.*, 2013). Priming is the process by which plants mount a stronger and faster immune response. The expression of *SBT3.3* is induced upon pathogen invasion, and *sbt3.3* mutants are more susceptible to *P. syringae* and *Hyaloperonospora arabidopsidis*. SBT3.3 primes plants for the transcriptional activation of defence-related genes following pathogen invasion by inducing chromatin remodelling in the form of activating histone marks at the promoters of SA-regulated defence-related genes including *PR1*, and at its own promoter, initiating a positive feedback loop (Ramírez *et al.*, 2013). However, the substrate and the mechanism of SBT3.3 activity remain enigmatic.

## Outlook

There is no doubt that proteases play important roles in plant immunity, and the emerging picture is that these roles are very diverse. Unravelling these different roles holds several major challenges.

The first challenge lies in unravelling a robust proteolytic network with intrinsic redundancies. Genetic redundancy improves the robustness of defence when multiple proteins, each with different sensitivities to, for example, pathogen-derived inhibitors, act on one or several substrates (Whitacre, 2012; Fares, 2015). A similar robust network has been described for diverse ‘helper’ and ‘sensor’ NLRs that confer immunity to a broad range of plant pathogens (Wu *et al.*, 2017). The expansion of the *P69* family in tomato (Jordá *et al.*, 2000; Reichardt *et al.*, 2018) as well as the clustering of papains in several plant families (Richau *et al.*, 2012) support the concept of selection for redundant protease networks. Compensation between different protease classes, such as Asp and Cys proteases, is also increasingly likely because redundant proteases may cleave at different sites within the same region of a substrate to produce the same outcome.

A second challenge is to understand how apoplastic proteases are regulated. Activation of apoplastic proteases may be dependent on endogenous regulators (Zimmermann *et al.*, 2016), proteolytic cascades (Paulus *et al.*, 2020), pH (Meyer *et al.*, 2016b), or redox status (Balakireva and Zamyatnin, 2019). Understanding how these elements coordinate protease function during pathogen invasion remains a challenge for future research.

The third, obvious challenge is the identification of biologically relevant substrates. Substrate identification techniques can return multiple candidates, but not all of these substrates may contribute to the observed immune phenotype. This can be tested experimentally, for instance using uncleavable mutant substrates. However, redundancy amongst substrates providing collective immunity may mask the role of individual substrates.

A fourth challenge is to determine if the prevalence of Cys and Ser proteases, particularly papains and subtilases, is due to their relative importance in immunity, or the result of a research bias, sparked by leading examples and supported by robust detection assays. Future efforts should also include unbiased approaches to identify secreted immune proteases and consider less well-characterized proteases.

In addition, we are left with several intriguing questions. For example, which apoplastic proteases are responsible for DAMP and PAMP release *in vivo*? For instance, proteases responsible for releasing the bacterial PAMPs from flagellin and EF-Tu remain to be identified. Furthermore, how do secreted proteases act collectively and consecutively on substrates? In addition, how do plant proteases distinguish between plant and pathogen substrates in order to prevent self-degradation? Finally, are extracellular protease repertoires different between plant species and is this important for co-evolution with other secreted host proteins? Or for being a non-host? These are just a few of the fascinating remaining questions waiting to be answered.
